# Identifying Relationships between *Glutathione S-Transferase-2* Single Nucleotide Polymorphisms and Hypoxia Tolerance and Growth Traits in *Macrobrachium nipponense*

**DOI:** 10.3390/ani14050666

**Published:** 2024-02-20

**Authors:** Xuanbin Gao, Zijian Gao, Minglei Zhang, Hui Qiao, Sufei Jiang, Wenyi Zhang, Yiwei Xiong, Shubo Jin, Hongtuo Fu

**Affiliations:** 1 Wuxi Fisheries College, Nanjing Agricultural University, Wuxi 214081, China; gaoxuanbin@163.com (X.G.); gaozijiangenomics@163.com (Z.G.); 2 Shandong Freshwater Fisheries Research Institute, Jinan 250013, China; zmlzzzqz@163.com; 3 Key Laboratory of Freshwater Fisheries and Germplasm Resources Utilization, Ministry of Agriculture, Freshwater Fisheries Research Center, Chinese Academy of Fishery Sciences, Wuxi 214081, China; qiaoh@ffrc.cn (H.Q.); jiangsf@ffrc.cn (S.J.); zhangwy@ffrc.cn (W.Z.); xiongyw@ffrc.cn (Y.X.)

**Keywords:** *Macrobrachium nipponense*, hypoxia, growth, *GST-2*, single nucleotide polymorphism

## Abstract

**Simple Summary:**

This study focused on investigating the genetic factors related to hypoxia tolerance and growth traits in *Macrobrachium nipponense*, a species of freshwater prawn. Specifically, a gene called glutathione S-transferase-2 (*GST-2*) and its single nucleotide polymorphism (SNP) in *M. nipponense* were studied. By analyzing samples from different populations, 38 SNP loci were identified in the *GST-2* gene. The genetic diversity of these loci was assessed, and associations between specific SNP loci and hypoxia tolerance and growth traits were studied. The results showed that certain SNP loci were significantly correlated with hypoxia tolerance and growth traits in different populations of *M. nipponense*. Moreover, some SNP loci associated with growth traits exhibited a high degree of genetic linkage. These findings provide valuable insights into the genetic improvement of growth and hypoxia tolerance in *M. nipponense*, which can contribute to the cultivation of prawns with desirable characteristics.

**Abstract:**

Investigating hypoxia tolerance and growth trait single nucleotide polymorphisms (SNPs) in *Macrobrachium nipponense* is conducive to cultivating prawns with hypoxia tolerance and good growth characteristics. The glutathione S-transferase-2 gene (*GST-2*) has been shown to regulate hypoxia responses in *M. nipponense*. In this study, we identified a single *GST-2* SNP in *M. nipponense*, and analyzed its regulatory relationship with hypoxia tolerance and growth. The *GST-2* sequence was amplified with a polymerase chain reaction from 197 “Taihu Lake No. 3”, “Taihu Lake No. 2”, and Pearl River population samples to identify SNP loci. The full-length *Mn-GST2* sequence was 2317 bp, including three exons and two introns. In total, 38 candidate SNP loci were identified from *GST-2* using Mega11.0 comparisons, with most loci moderately polymorphic in terms of genetic diversity. Locus genotypes were also analyzed, and basic genetic parameters for loci were calculated using Popgene32 and *PIC_CALC*. The expected heterozygosity of the 38 SNP loci ranged from 0.2334 to 0.4997, with an average of 0.4107, while observed heterozygosity ranged from 0.1929 to 0.4721, with an average of 0.3401. The polymorphic information content ranged from 0.21 to 0.37. From SPSS analyses, the G+256A locus was significantly correlated with hypoxia tolerance across all three *M. nipponense* populations, while the SNP loci A+261C, C+898T, A+1370C, and G+1373T were significantly associated with growth traits. Further analyses revealed that the T+2017C locus was significantly correlated with hypoxia tolerance in “Taihu Lake No. 2” populations, G+256A, A+808T, C+1032T, and A+1530G loci were significantly correlated with hypoxia tolerance in “Taihu Lake No. 3” populations, while no SNP loci were correlated with hypoxia tolerance in Pearl River populations. A+1370C and G+1373T loci, which were associated with growth traits, exhibited a high degree of linkage disequilibrium (r^2^ = 0.89 and r^2^ > 0.8), suggesting potential genetic linkage. Our data suggest associations between hypoxia tolerance and growth trait SNP loci in *M. nipponense*, and provide valuable evidence for the genetic improvement of growth and hypoxia tolerance in this prawn species.

## 1. Introduction

The Oriental river prawn, *Macrobrachium nipponense* (Crustacea; Decapoda; Palaemonidae), is widely distributed across China and other Asian countries [1], and are widely cultured in southern regions of China due to their desirable meat quality and high nutritional value [2]. This approach also provides high-quality food and huge economic benefits to local farmers and consumers. The prawn is an important economic aquaculture species, with annual production levels of more than 250,000 tons and an output of over 2 billion RMB in China. However, a major issue with these prawns is their hypoxia-sensitive characteristics, which affect large-scale aquaculture production [3]. Hypoxic conditions cause stress, which essentially disturbs optimal crustacean development, causing reduced molting frequencies, evasive behaviors, slow growth, suppressed immune functions, and even death [4,5]. Therefore, the molecular mechanisms underlying hypoxia responses in *M. nipponense* must be investigated. [6]. In aquatic environments, oxygen levels often fluctuate, and crustaceans frequently experience hypoxic conditions [7]. Previous studies reported that the lethal concentration 50 oxygen value at 8 h was only 0.85 mg/L in *M. nipponense* [8], which is dramatically lower than the other crustacean species [9]. However, the molecular basis underpinning this chronic hypoxia exposure remains poorly characterized, with chronic hypoxia a major physiological challenge for prawns in culture [10]. Previous studies showed that glutathione S-transferase-2 (*GST-2*) is a hypoxia-inducible factor in *M. nipponense*, and helps regulate oxidative stress under hypoxia conditions [9].

*GSTs* are a multifunctional protein superfamily with essential roles in many physiological functions across a wide variety of organisms, including xenobiotic detoxification, protection against oxidative damage, and hormone and exogenous chemical intracellular transport [11,12]. *GST* enables the tripeptide glutathione (*GSH*) to catalyze the nucleophilic attack on electrophilic groups of hydrophobic toxic compounds, promoting their excretion from the cell [13]. The *GST* antioxidant system has essential roles in cellular defenses against reactive free radicals and other oxidants [14,15], which are also antioxidant enzymes involved in the regulation of stress responses, drug resistance, cell proliferation and death [13]. Previous studies have reported two *GSTs* isoforms in *M. nipponense* hepatopancreas (*GST-1* and *GST-2*); *GST-2* appears to have more important roles in the hepatopancreas and gills than *GST-1* during chronic hypoxia stress [9].

The identification of molecular DNA markers is critical in animal genetics research [16]. Such markers are widely used to in population genetics and phylogenic analyses [17,18,19]. Single nucleotide polymorphisms (SNPs) represent a third-generation molecular marker technology [20]; they are used to improve growth traits and disease resistance in aquatic animals [21], and provide a theoretical basis for breeding high-quality aquatic animals.

In this study, we identified *GST-2* SNP loci in *M. nipponense* and analyzed correlations with hypoxia tolerance and growth traits. These findings provide potential candidate markers for genetically improving hypoxia tolerance and growth traits in *M. nipponense*, with a view to promoting sustainable aquaculture development and large-scale prawn breeding.

## 2. Methods and Materials

### 2.1. M. nipponense Origin and Experimantal Design

All *M. nipponense* used in the present study were collected from the same pond cultured in Dapu scientific experimental base in Freshwater Fisheries Research Center (Wuxi, China)*. M. nipponense* samples were collected from three different areas (“Taihu Lake No. 2”, “Taihu Lake No. 3”, and Pearl River), which were hatched in May 2023, and cultured for approximately 4 months. Each population contained 100 individuals in 3 different tanks. Before hypoxia treatments, average body weights of “Taihu Lake No. 2”, “Taihu Lake No. 3”, and Pearl River populations were 1.18 ± 0.76 g, 2.53 ± 1.20 g, and 1.30 ± 0.82 g, respectively. Average body lengths of “Taihu Lake No. 2”, “Taihu Lake No. 3”, and Pearl River samples were 19.03 ± 5.13 mm, 23.37 ± 5.21 mm, and 20.00 ± 5.06 mm, respectively. Average total lengths of “Taihu Lake No. 2”, “Taihu Lake No. 3”, and Pearl River samples were 41.76 ± 10.17 mm, 53.32 ± 11.86 mm, and 43.26 ± 11.65 mm, respectively. All prawns were maintained under laboratory conditions (21.0 °C ± 0.5 °C, pH 8.2 ± 0.08) for 72 h with a dissolved oxygen content of 6.5 mg/L prior to hypoxia treatments. After an acclimation period, the oxygen concentration was reduced to 0.85 ± 0.05 mg/L by adding N_2_ to the water [9]. N_2_ solubility in water is relatively low; introducing N_2_ into a body of water gradually displaces dissolved oxygen and reduces concentrations. Levels were measured every 2 h using a Portable DO Meter (Beyotime, Shanghai, China). Critically, if dissolved oxygen levels were <0.85 mg/L, oxygen was pumped into tanks to maintain 0.85 mg/L levels. Hypoxia treatments were maintained for 8 h and individual death times, total length, body length, and body weight recorded. Dead prawns were recorded immediately, which reduced errors. After hypoxia treatments, surviving prawns were reoxygenated for 4 h before collection. In total, 31 survivors were randomly collected from the “Taihu Lake No. 2” and Pearl River populations, while all remaining 37 survivors were collected from the “Taihu Lake No. 3” population. Total length, body length, and body weight of survivors were recorded. It was hypothesized that survivors may have had a higher tolerance to hypoxia than dead individuals. Finally, 207 prawns were collected, including 49 individuals from the Pearl River, 58 from “Taihu Lake No. 2”, and 100 from “Taihu Lake No. 3”, which amounted to 99 survivors and 108 dead prawns. Muscle tissues were immediately removed and snap frozen in liquid nitrogen to prevent DNA degradation and stored at −80 °C until DNA extraction.

### 2.2. Genomic DNA Extraction and Cloning of the GST-2 Gene

Muscle DNA was extracted using the Takara DNA extraction kit (Takara Bio, Inc., Otsu, Shiga, Japan), following manufacturer’s instructions. DNA quality was determined using 1.2% agarose gel electrophoresis, and DNA concentrations measured using an ultraviolet spectrophotometer (Eppendorf, Germany). Extracted DNA was stored at −20 °C for *GST-2* SNP loci identification. The genome sequence of *GST-2* gene of *M. nipponense* registered in the NCBI database was used for primer designing (GenBank Accession Number: MG787173, https://ftp.cngb.org/pub/CNSA/data2/CNP0001186/CNS0254395/CNA0014632/ (accessed on 27 May 2023)). Four full *GST-2* sequence primer pairs (Table 1) were designed using primer premier 5.0 [22], and polymerase chain reaction (PCR) was used to amplify DNA fragments with primers (Shanghai, China). PCR products were assessed using 1.2% agarose gel electrophoresis, and pair-end sequenced by Shanghai Shenggong Bioengineering Technology Service Co., LTD (Shanghai, China) on an ABI3730 automated sequencer (Invitrogen Biotechnology Co., Ltd., Carlsbad, CA, USA) [23].

### 2.3. SNPs Identification and Association Analysis

Sequencing results were aligned using Mega 11.0 software [24] to identify *GST-2* SNP loci. Genetic parameters, including effective allele number (Ne), observed heterozygosity (Ho), and expected heterozygosity (He), were calculated using Popgene32 software [25]. Additionally, polymorphic information content (PIC) was calculated using *PIC_CALC* [26]. SPSS 23.0 was used to analyze correlations between *GST-2* SNP loci and hypoxia tolerance and growth traits [27]. Correlations with average survival times under hypoxia were analyzed using Kaplan–Meier regression in Survival model. This model is generally used to analyze individuals who survived and died. The variable “Time” represented prawn survival times; a “0” value indicated a dead prawn and “1” indicated a survivor. Fixed factors were SNP loci genotypes. Correlations with growth traits were analyzed using multivariate analysis in the general linear model. Fixed factors were SNP loci genotypes, while dependent variables were growth traits. A *p* < 0.05 value indicated a significant correlation.

### 2.4. Linkage Disequilibrium (LD) Analysis of GST-2 SNPs

An LD analysis of the 38 *GST-2* SNP loci was conducted using default parameters in the R language package Gaston. An r^2^ value = 0 indicated complete linkage equilibrium, while 1 represented complete LD. An r^2^ > 0.8 value indicated a strong LD, while an r^2^ < 0.3 value indicated a weak LD [28].

## 3. Result

### 3.1. Survival Comparisons between Different M. nipponense Populations

*M. nipponense* death times and numbers across the three populations are shown (Table 2) after an 8 h treatment in a hypoxia environment. In total, 63, 27, and 18 prawns were dead and collected from “Taihu Lake No. 3”, “Taihu Lake No. 2”, and Pearl River populations, respectively. The “Taihu Lake No. 3” population had the highest number of deaths, while the Pearl River population had the lowest. The Pearl River population also showed the strongest ability to resist hypoxia when compared to “Taihu Lake No. 3” and “Taihu Lake No. 2” populations.

### 3.2. Gene Structure and GST-2 SNP Identification

In total, ten samples (three from Pearl River, six from “Taihu Lake No. 2”, and one from “Taihu Lake No. 3” populations) failed sequencing and were removed from analyses. Therefore, 197 samples were included, comprising 29 survivors and 17 dead individuals from the Pearl River population, 28 survivors and 24 dead individuals from “Taihu Lake No. 2”, and 37 survivors and 62 dead individuals from “Taihu Lake No. 3”. Therefore, 103 dead individuals and 94 survivors were included. The *M. nipponense GST-2* genome sequence was obtained from the *M. nipponense* genome and verified using four primer pairs. The full-length *Mn*-*GST2* sequence was 2317 bp, including three exons and two introns. The exon region was 660 bp long and the intron region was 1657 bp. Four primer pairs were used to amplify DNA fragments in samples. Loci with a minimum allele frequency > 10% were deemed sequencing errors and discarded. Finally, 38 high-quality SNP loci were obtained and named based on the genomic sequence starting from the initiation codon (ATG) to the mutated base position. Among SNP loci, C+898T, C+1003T, C+2002A, T+2017C, and A+2183G were exon mutations (non-synonymous mutations). The TGG codon was changed to CGG, resulting in a tryptophan substitution to arginine at the C+898T locus. The TTT codon was changed to CTT, leading to a phenylalanine replacement with leucine at the C+1003T locus. The ACC codon was changed to CCC, resulting in a threonine substitution to proline at the C+2002A locus. The CAA codon was changed to TAA, leading to a glutamine replacement with glycine at the T+2017C locus. The CGT codon was changed to CAT, resulting in an arginine substitution to histidine at the A+2183G locus. The remaining SNPs were intronic mutations.

### 3.3. SNPs Polymorphism of GST-2 Gene in M. nipponense

The genetic parameters of *GST-2* SNP polymorphisms were analyzed using Popgene32 (Table 3). The He of the 38 SNP loci ranged from 0.2334 to 0.4997 (average = 0.4107), while the Ho ranged from 0.1929 to 0.4721 (average = 0.3401). PIC values ranged from 0.21 to 0.37. PIC values for C+820T, G+1544A, and T+2017C were < 0.25, and PIC values for the remaining SNP loci were from 0.27 to 0.37. No SNP loci had PIC values > 0.5. The Ne was 1.7130, and the average genetic diversity index (Nei) was 0.4096. These values indicated that our *M. nipponense* population exhibited a relatively high level of genetic diversity.

### 3.4. Correlation Analysis of GST-2 SNPs and Hypoxia Tolerance Traits in M. nipponense

In previous hypoxia treatments, surviving prawns were reoxygenated for 4 h before collection, and the survival times were recorded for 12 h. In our study, genotype frequencies for the 38 SNP loci in surviving and dead prawns were calculated (Table 4). Only the locus G+256A genotype showed significant differences between surviving and dead prawns. At this locus, the highest frequency of the AA genotype in dead individuals was 48.5%, while AG and GG genotype frequencies were only 23.3% and 28.2%, respectively. The highest GG genotype frequency in survivors was 41.5%, while AG and AA genotype frequencies were only 27.7% and 30.9%, respectively. The GG genotype frequency in survivors was significantly higher when compared with dead prawns. In the present study, the genotype frequency of these 38 SNP loci in surviving prawns and dead prawns were calculated, which was presented in Table 4.

Potential relationships between G+256A and hypoxia tolerance were analyzed across all samples using SPSS 23.0 (Table 5). The AA genotype frequency was 40.1%, with an average survival time = 725.90 ± 62.25 min. The GG genotype frequency was 34.5%, with an average survival time = 964.40 ± 67.75 min, showed significant difference with the average survival time corresponding to the AA genotype (*p* < 0.05). The GG genotype corresponded to the longest average survival times, followed by the AG genotype (average survival times), while the AA genotype corresponded to the shortest average survival times.

Potential relationships between SNPs and hypoxia tolerance were analyzed using SPSS 23.0. No SNP loci related to hypoxia were identified in the Pearl River population, while the T+2017C locus exhibited a potential relationship with hypoxia in the “Taihu Lake No. 2” population (Table 6), and G+256A, A+808T, C+1032T, and A+1530G loci demonstrated potential relationships with hypoxia in the “Taihu Lake No. 3” population (Table 7). As indicated in Table 6, the TT genotype frequency at the T+2017C locus was 5.8%, with an average survival time = 396.67 ± 3.33 min in 52 “Taihu Lake No. 2” individuals, while the CC genotype frequency was 86.5%, with an average survival time of 975.60 ± 85.68 min, and showed significant differences with TT and CT genotypes (*p* < 0.05). As shown in Table 7, associations between SNPs and hypoxia were identified in 99 individuals from “Taihu Lake No. 3”. In this table, the AA genotype frequency at the G+256A locus was 45.5%, with an average survival time = 562.82 ± 67.02 min, while the GG genotype frequency was 35.4%, with an average survival time of 948.54 ± 91.69 min (*p* < 0.05). The AT genotype frequency at the A+808T locus was 16.2%, with an average survival time = 473.31 ± 96.25 min, while the TT genotype frequency was 66.7%, with an average survival time = 855.67 ± 68.52 min (*p* < 0.05). The CT genotype frequency at the C+1032T locus was 18.2%, with an average survival time = 520.50 ± 100.21 min, while the TT genotype frequency was 72.7%, with an average survival time = 830.99 ± 65.06 min, and showed significant differences with CC and CT genotypes (*p* < 0.05). The AG genotype frequency at the A+1530G locus was 20.2%, with an average survival time = 360.30 ± 29.38 min, while the GG genotype frequency was 71.7%, with an average survival time = 863.16 ± 66.34 min (*p* < 0.05).

### 3.5. Correlations between GST-2 SNPs and Growth Traits

Total length, body weight, and body length values are key economic traits which have positive effects on *M. nipponense* quality and yield. Association analyses between 38 SNP loci and growth traits were also performed using SPSS 23.0 (Table 8). Significant differences (*p* < 0.05) were observed at seven SNP loci for total length, thirteen SNP loci for body weight, and four SNP loci for body length. Among loci, four were simultaneously corelated with total length, body weight, and body length: A+261C, C+898T, A+1370C, and G+1373T loci. The AC genotype at the A+261C locus, the TT genotype at the C+898T locus, the CC genotype at the A+1370C locus, and the TG genotype at the G+1373T locus were significantly associated with growth traits and showed better growth performances than the other genotypes (*p* < 0.05).

### 3.6. LD Analysis of GST-2 SNPs

**Figure 1:**
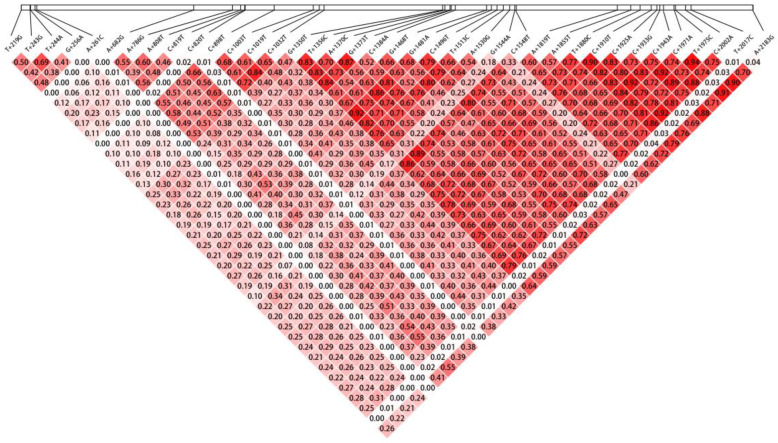
The r^2^ values for linkage disequilibrium analysis of the SNP loci in *GST-2* gene of *M. nipponense*.

An LD analysis of the 38 *GST-2* SNP loci was performed in the R software package Gaston (v1.5.9) (Figure 1). Color shading in the figure reflected the magnitude of r^2^ values, which were used to measure non-random associations between two SNP loci. An r^2^ value = 0 indicated complete linkage equilibrium, while an r^2^ value = 1 indicated complete LD. The r^2^ ranges were from 0 to 0.94. In total, 33 pairs exhibited strong LD (r^2^ > 0.8), indicating a high degree of correlation between SNP loci. Additionally, 278 pairs exhibited weak LD (r^2^ < 0.3), indicating lower correlation levels. In particular, a significant association was observed between C+1971A and T+1975C (highest r^2^ value), suggesting a potentially high degree of LD. Biologically, if SNPs were contained on exons, they were more likely to move together as a functional protein sequence.

## 4. Discussion

### 4.1. Hypoxia Tolerance and Growth Performance in M. nipponense Populations

Currently, the main issue in the *M. nipponense* aquaculture industry is low hypoxia tolerance, which restricts sustainable industry development. In our study, we sought to identify *GST-2* SNP loci, which were reportedly involved in hypoxia tolerance in *M. nipponense* [9]. Correlations between SNP loci and hypoxia tolerance and growth traits were analyzed in 197 samples from “Taihu Lake No. 3”, “Taihu Lake No. 2”, and Pearl River populations. “Taihu Lake No. 2” and “Taihu Lake No. 3” populations are new *M. nipponense* varieties generated via hybridization and family selection, respectively, and have shown better growth performances than wild populations. Pearl River populations have shown better growth and greater tolerance levels to hypoxia than other wild populations, and have been used as a base population for new breeding improvements in our laboratory. As indicated (Table 2), dead prawn numbers in the Pearl River population were significantly lower than in “Taihu Lake No. 2” and “Taihu Lake No. 3” populations. Prawn body weights in the “Taihu Lake No. 3” population were almost twice that of “Taihu Lake No. 2” and Pearl River populations. These data indicated that prawns from “Taihu Lake No. 3” showed better growth performances than those in “Taihu Lake No. 2” and Pearl River. Body weights were shown to have negative effects on hypoxia tolerance, consistent with previous research [29].

### 4.2. The Identification and Polymorphism Analysis of GST-2 SNP Loci

Selected SNP markers from target genes are critical for marker-assisted breeding and are used to improve genetic accuracy and efficiency in aquatic animals [30]. SNPs in target genes are often closely associated with essential biological traits, such as growth, disease resistance, and immune responses [31,32]. Currently, SNP studies in aquatic animals have predominantly focused on growth traits; previous studies successfully identified growth-related SNP loci in several shrimp species, including *M. japonicus* [33], *M. rosenbergii* [34], and *Litopenaeus vannamei* [35]. Previous studies also reported that lactate dehydrogenase [36], glutathione peroxidase [37], *p53* [38], *GST* [9], and caspase-3 [39] were involved in hypoxia tolerance in *M. nipponense*. *GST-2* is an important detoxification enzyme, with key roles in responses to hypoxia [40]. In our study, full-length *GST-2* measured 2317 bp, with 38 high-quality SNP loci identified. The gene frequencies of these 38 loci were compared across three populations, with no significant differences (*p* > 0.05) recorded. In loci, the principal component analysis was carried out using the R package. No clear clustering was observed across populations, indicating the absence of significant population structures. Therefore, no population structure adjustments were performed. The SNP distribution frequency for *GST-2* was 1.64/100 bp. It was previously reported that SNP distribution frequencies varied across different species, such as 0.15/100 bp in *Portunus trituberculatus*, 0.19/100 bp in *Physeter macrocephalus*, and 0.20/100 bp in *Cynoglossus semilaevis* [41,42,43]. The *GST-2* SNP distribution frequency in *M. nipponense* was significantly higher than in other species, and was probably due to long intron sequences. In total, 38 SNPs were identified with an average Ne value = 1.7130, indicating a uniform SNP loci distribution. Average He and PIC values were 0.3401 and 0.3236, respectively. A locus is considered highly polymorphic when the PIC value is >0.5, moderately polymorphic when the PIC value is between 0.25 and 0.5, and lowly polymorphic at PIC < 0.25 [44]. In our study, the majority of SNP loci were moderately polymorphic, except for C+820T, G+1544A, and T+2017C loci, which were poorly polymorphic. However, no highly polymorphic SNP loci were observed, suggesting that the majority of SNPs were moderately polymorphic and provided sufficient information for marker-assisted *M. nipponense* breeding.

### 4.3. Correlations between SNP Loci and Hypoxia Tolerance and Growth Traits

SNPs widely occur in the genomes of various organisms, with mutations in coding regions causing changes in protein structure and function [28,45]. Although introns are traditionally considered non-functional and unrelated sequences for amino acid translation, recent studies have reported that intronic mutations can disrupt transcription factor binding and impact splice site integrity during transcription processes, thereby causing functional abnormalities in protein products [45,46,47,48,49]. The *GST* G+256A locus in *M. nipponense* is an intronic mutation; the GG genotype at G+256A showed longer survival times than other genotypes, suggesting that the G allele at this locus may make *M. nipponense* more tolerant to hypoxia. The CC genotype at the T+2017C locus in “Taihu Lake No. 2” samples showed longer survival times than the other genotypes, suggesting that the C allele at this locus may make “Taihu Lake No. 2” prawns more tolerant to hypoxia. GG, TT, TT, and GG genotypes at G+256A, A+808T, C+1032T, and A+1530G loci in “Taihu Lake No. 3” samples, respectively, showed longer survival times than the other genotypes. These results suggested that G, T, T, and G alleles at G+256A, A+808T, C+1032T, and A+1530G loci, respectively, may have contributed to higher hypoxia tolerance in “Taihu Lake No. 3” samples. A+261C, C+898T, A+1370C, and G+1373T loci were simultaneously corelated with total length, body weight, and body length, and A, T, C, and T alleles at A+261C, C+898T, A+1370C, and G+1373T loci, respectively, may have promoted genetic improvements in *M. nipponense* growth performances.

### 4.4. LD Analysis

LD is a useful approach to measure non-random allele associations at different loci in a given population [28]. In our study, LD was represented by r^2^ values, which provided a more reliable measurement than that of D’, independent of the sample size [50]. LD depends on at least two factors: the recombination frequency and the number of generations since a mutation was introduced into a population. If two loci are physically close to each other, or if the genetic recombination frequency between loci is low, then they are more likely to have a strong LD [51]. A+1370C and G+1373T loci, which were associated with growth traits, exhibited a high LD (r^2^ = 0.89 and r^2^ > 0.8, respectively), suggesting potential genetic linkage. This may have benefitted the cultivation of individuals with good growth traits, and could facilitate the genetic and evolutionary analyses of the species. Genetic diversity is important for a species’ adaptation to new environments; genetic diversity in *M. nipponense* provided a broader range of genetic variations in populations. Such diversity increases a species’ ability to cope with environmental challenges [51].

## 5. Conclusions

We successfully identified *GST-2* SNP loci correlations with hypoxia tolerance and growth traits in *M. nipponense*. The G+256A locus may be used for the molecular marker-assisted selection of hypoxia tolerance in these prawns. The T+2017C loci may be used for the molecular marker-assisted selection of hypoxia tolerance in “Taihu Lake No.2” prawns. A+808T, C+1032T, and A+1530G loci may be used for the molecular marker-assisted selection of hypoxia tolerance in “Taihu Lake No. 3” prawns. Also, A+261C, C+898T, A+1370C, and G+1373T loci may be useful markers to genetically improve growth traits. In future work, SNP loci will be characterized from other hypoxia-related genes to improve the genetic accuracy and efficiency of hypoxia tolerance in *M. nipponense*.

## Figures and Tables

**Table 1:** Primers designed based on the full *GST-2* sequence were used for PCR.

Primer	Primer Sequence (5′-3′)	Amplification Length (bp)
GST-1F	ATGCCCCTCGATCTCTACTAC	287
GST-1R	GTTGTCACTTGTTGTTAATAAG	
GST-2F	CAACTCACTGAATTCTTTCAGG	581
GST-2R	GAATCGACCTGAATTGTCCG	
GST-3F	GTTCTGCGTCATCCTATATTCG	443
GST-3R	GTCTCTTAGATGAACTTCAAGC	
GST-4F	ATCCAAGGACGATAACTTTGAGA	526
GST-4R	TGATCATCGACCTCACGAAATCA	

**Table 2:** Death statistics of different populations at different death times.

Population	0–2 h	2–4 h	4–6 h	6–8 h	Total
“Taihu Lake No. 3”	2	11	22	28	63
“Taihu Lake No. 2”	6	5	8	8	27
“Pearl River”	3	5	8	2	18

**Table 3:** Genetic polymorphism of Single Nucleotide Polymorphisms (SNPs) in *M. nipponense*.

Serial Number	SNP Locus	Effective Number of Alleles (Ne)	Observed Heterozygosity (Ho)	Expected Heterozygosity (He)	Genetic Diversity Index (Nei)	Polymorphic Information Content (PIC)
1	T+219G	1.9246	0.1929	0.4816	0.4804	0.37
2	T+243G	1.9900	0.2183	0.4987	0.4975	0.37
3	T+244A	1.9938	0.2538	0.4997	0.4984	0.37
4	G+256A	1.9938	0.2538	0.4997	0.4984	0.37
5	A+261C	1.8198	0.5939	0.4516	0.4505	0.35
6	A+682G	1.5967	0.2335	0.3747	0.3737	0.30
7	A+786G	1.7871	0.3299	0.4415	0.4404	0.34
8	A+808T	1.7643	0.2487	0.4343	0.4332	0.34
9	C+819T	1.4587	0.1980	0.3153	0.3145	0.27
10	C+820T	1.3162	0.1878	0.2408	0.2402	0.21
11	C+898T	1.5772	0.2386	0.3669	0.3660	0.30
12	C+1003T	1.5115	0.2284	0.3392	0.3384	0.28
13	C+1019T	1.5115	0.2589	0.3392	0.3384	0.28
14	C+1032T	1.5181	0.2538	0.3421	0.3413	0.28
15	G+1350T	1.7527	0.4315	0.4305	0.4294	0.34
16	T+1356C	1.7229	0.4264	0.4207	0.4196	0.33
17	A+1370C	1.7468	0.4162	0.4286	0.4275	0.34
18	G+1373T	1.6923	0.3909	0.4101	0.4091	0.33
19	C+1384A	1.8405	0.3604	0.4578	0.4567	0.35
20	G+1468T	1.9050	0.4721	0.4763	0.4751	0.36
21	G+1481A	1.7585	0.4162	0.4324	0.4313	0.34
22	C+1496T	1.6673	0.3807	0.4012	0.4002	0.32
23	T+1513C	1.8554	0.4365	0.4622	0.4610	0.35
24	A+1530G	1.5772	0.3096	0.3669	0.3660	0.30
25	G+1544A	1.3480	0.2030	0.2588	0.2582	0.22
26	C+1548T	1.7290	0.4518	0.4227	0.4216	0.33
27	A+1819T	1.7169	0.3299	0.4186	0.4175	0.33
28	A+1855T	1.8650	0.3858	0.4650	0.4638	0.36
29	T+1880C	1.7527	0.3807	0.4305	0.4294	0.34
30	C+1910T	1.7349	0.3959	0.4247	0.4236	0.33
31	C+1925A	1.6799	0.3503	0.4057	0.4047	0.32
32	C+1933G	1.7926	0.4264	0.4433	0.4422	0.34
33	C+1943A	1.7169	0.3909	0.4186	0.4175	0.33
34	C+1971A	1.7982	0.4315	0.4450	0.4439	0.35
35	T+1975C	1.8144	0.4264	0.4500	0.4489	0.35
36	C+2002A	1.7290	0.4112	0.4227	0.4216	0.33
37	T+2017C	1.3035	0.1675	0.2334	0.2328	0.21
38	A+2183G	1.8302	0.4416	0.4548	0.4536	0.35
	Average	1.7130	0.3401	0.4107	0.4096	0.32

**Table 4:** Genotyping frequencies ratio of the 38 SNP loci in the deceased and surviving *M. nipponense*.

Locus	Genotype	Genotype Frequency Ratio of Deceased *M. nipponense*	Genotype Frequency Ratio of Surviving *M. nipponense*
T+219G	GG: GT: TT	31 (0.301): 15 (0.146): 57 (0.553)	29 (0.309): 23 (0.245): 42 (0.447)
T+243G	GG: GT: TT	40 (0.388): 17 (0.165): 46 (0.447)	30 (0.319): 26 (0.277): 38 (0.404)
T+244A	AA: AT: TT	40 (0.388): 25 (0.243): 38 (0.369)	39 (0.415): 25 (0.266): 30 (0.319)
G+256A	AA: AG: GG	50 (0.485): 24 (0.233): 29 (0.282)	29 (0.309): 26 (0.277): 39 (0.415)
A+261C	AA: AC: CC	8 (0.078): 62 (0.602): 33 (0.32)	1 (0.011): 55 (0.585): 38 (0.404)
A+682G	AA: AG: GG	13 (0.126): 26 (0.252): 64 (0.621)	13 (0.138): 20 (0.213): 61 (0.649)
A+786G	AA: AG: GG	18 (0.175): 31 (0.301): 54 (0.524)	14 (0.149): 34 (0.362): 46 (0.489)
A+808T	AA: AT: TT	20 (0.194): 26 (0.252): 57 (0.553)	18 (0.191): 23 (0.245): 53 (0.564)
C+819T	CC: CT: TT	8 (0.078): 21 (0.204): 74 (0.718)	11 (0.117): 18 (0.191): 65 (0.691)
C+820T	CC: CT: TT	4 (0.039): 21 (0.204): 78 (0.757)	5 (0.053): 16 (0.17): 73 (0.777)
C+898T	CC: CT: TT	11 (0.107): 27 (0.262): 65 (0.631)	13 (0.138): 20 (0.213): 61 (0.649)
C+1003T	CC: CT: TT	10 (0.097): 26 (0.252): 67 (0.65)	10 (0.106): 19 (0.202): 65 (0.691)
C+1019T	CC: CT: TT	7 (0.068): 31 (0.301): 65 (0.631)	10 (0.106): 20 (0.213): 64 (0.681)
C+1032T	CC: CT: TT	9 (0.087): 28 (0.272): 66 (0.641)	9 (0.096): 22 (0.234): 63 (0.67)
G+1350T	GG: GT: TT	9 (0.087): 46 (0.447): 48 (0.466)	10 (0.106): 39 (0.415): 45 (0.479)
T+1356C	CC: CT: TT	51 (0.495): 43 (0.417): 9 (0.087)	45 (0.479): 41 (0.436): 8 (0.085)
A+1370C	AA: AC: CC	11 (0.107): 40 (0.388): 52 (0.505)	9 (0.096): 42 (0.447): 43 (0.457)
G+1373T	GG: GT: TT	9 (0.087): 39 (0.379): 55 (0.534)	9 (0.096): 38 (0.404): 47 (0.5)
C+1384A	AA: AC: CC	48 (0.466): 36 (0.35): 19 (0.184)	44 (0.468): 35 (0.372): 15 (0.16)
G+1468T	GG: GT: TT	16 (0.155): 46 (0.447): 41 (0.398)	14 (0.149): 47 (0.5): 33 (0.351)
G+1481A	AA: AG: GG	50 (0.485): 42 (0.408): 11 (0.107)	44 (0.468): 40 (0.426): 10 (0.106)
C+1496T	CC: CT: TT	8 (0.078): 38 (0.369): 57 (0.553)	9 (0.096): 37 (0.394): 48 (0.511)
T+1513C	CC: CT: TT	44 (0.427): 43 (0.417): 16 (0.155)	39 (0.415): 43 (0.457): 12 (0.128)
A+1530G	AA: AG: GG	8 (0.078): 39 (0.379): 56 (0.544)	9 (0.096): 22 (0.234): 63 (0.67)
G+1544A	AA: AG: GG	77 (0.748): 21 (0.204): 5 (0.049)	70 (0.745): 19 (0.202): 5 (0.053)
C+1548T	CC: CT: TT	7 (0.068): 46 (0.447): 50 (0.485)	8 (0.085): 43 (0.457): 43 (0.457)
A+1819T	AA: AT: TT	15 (0.146): 30 (0.291): 58 (0.563)	11 (0.117): 35 (0.372): 48 (0.511)
A+1855T	AA: AT: TT	48 (0.466): 42 (0.408): 13 (0.126)	39 (0.415): 34 (0.362): 21 (0.223)
T+1880C	CC: CT: TT	49 (0.476): 41 (0.398): 13 (0.126)	49 (0.521): 34 (0.362): 11 (0.117)
C+1910T	CC: CT: TT	11 (0.107): 43 (0.417): 49 (0.476)	10 (0.106): 35 (0.372): 49 (0.521)
C+1925A	AA: AC: CC	59 (0.573): 34 (0.33): 10 (0.097)	48 (0.511): 35 (0.372): 11 (0.117)
C+1933G	CC: CG: GG	12 (0.117): 44 (0.427): 47 (0.456)	11 (0.117): 40 (0.426): 43 (0.457)
C+1943A	AA: AC: CC	52 (0.505): 41 (0.398): 10 (0.097)	48 (0.511): 36 (0.383): 10 (0.106)
C+1971A	AA: AC: CC	48 (0.466): 43 (0.417): 12 (0.117)	41 (0.436): 42 (0.447): 11 (0.117)
T+1975C	CC: CT: TT	47 (0.456): 44 (0.427): 12 (0.117)	41 (0.436): 40 (0.426): 13 (0.138)
C+2002A	AA: AC: CC	49 (0.476): 44 (0.427): 10 (0.097)	48 (0.511): 37 (0.394): 9 (0.096)
T+2017C	CC: CT: TT	78 (0.757): 19 (0.184): 6 (0.058)	76 (0.809): 14 (0.149): 4 (0.043)
A+2183G	AA: AG: GG	12 (0.117): 43 (0.417): 48 (0.466)	13 (0.138): 44 (0.468): 37 (0.394)

Note: The number and proportion of genotypes for each SNP locus in two populations: deceased and surviving. The numbers outside the parentheses represent the genotype counts, while the numbers inside the parentheses represent the proportions of each genotype.

**Table 5:** Average survival time of G+256A locus in the *M. nipponense*.

SNP Locus	Genotype	Sample Number	Genotype Frequency	Allele Frequency	Average Survival Time/Min
G+256A	AA	79	0.401	A/0.528	725.90 ± 62.25 ^a^
	AG	50	0.254	G/0.472	887.84 ± 81.95 ^ab^
	GG	68	0.345		964.40 ± 67.75 ^b^

Note: Mean values in the same column with different superscripts (a, b) are significantly different (*p* < 0.05).

**Table 6:** Average survival time of T+2017C locus in the “Taihu Lake No. 2”.

SNP Locus	Genotype	Sample Number	Genotype Frequency	Allele Frequency	Average Survival Time/Min
T+2017C	CC	45	0.865	C/0.90	975.60 ± 85.68 ^b^
	CT	4	0.077	T/0.10	468.75 ± 281.12 ^a^
	TT	3	0.058		396.67 ± 3.33 ^a^

Note: Mean values in the same column with different superscripts (a, b) are significantly different (*p* < 0.05).

**Table 7:** Average survival time of loci in the “Taihu Lake No. 3”.

SNP Locus	Genotype	Sample Number	Genotype Frequency	Allele Frequency	Average Survival Time/Min
G+256A	AA	45	0.455	A/0.551	562.82 ± 67.02 ^a^
	AG	19	0.192	G/0.449	832.16 ± 133.90 ^ab^
	GG	35	0.354		948.54 ± 91.69 ^b^
A+808T	AA	17	0.172	A/0.253	605.29 ± 114.86 ^ab^
	AT	16	0.162	T/0.747	473.31 ± 96.25 ^a^
	TT	66	0.667		855.67 ± 68.52 ^b^
C+1032T	CC	9	0.091	C/0.182	570.78 ± 158.95 ^a^
	CT	18	0.182	T/0.818	520.50 ± 100.21 ^a^
	TT	72	0.727		830.99 ± 65.06 ^b^
A+1530G	AA	8	0.081	A/0.182	730.88 ± 197.19 ^b^
	AG	20	0.202	G/0.818	360.30 ± 29.38 ^a^
	GG	71	0.717		863.16 ± 66.34 ^b^

Note: Mean values in the same column with different superscripts (a, b) are significantly different (*p* < 0.05).

**Table 8:** SNPs significantly associated with growth traits.

SNP Locus	Genotype	Sample Number	Total Length/mm	Weight/g	Body Length/mm
A+261C	AA	9	46.73 ± 15.91 ^ab^	1.90 ± 1.51 ^ab^	20.15 ± 6.99
	AC	117	49.90 ± 12.45 ^b^	2.11 ± 1.23 ^b^	22.25 ± 5.36
	CC	71	44.81 ± 11.87 ^a^	1.52 ± 1.05 ^a^	20.26 ± 5.35
C+898T	CC	24	47.06 ± 11.08 ^ab^	1.67 ± 0.93 ^ab^	21.13 ± 4.99 ^ab^
	CT	47	43.67 ± 12.31 ^a^	1.51 ± 1.17 ^a^	19.69 ± 5.50 ^a^
	TT	126	49.66 ± 12.64 ^b^	2.07 ± 1.23 ^b^	22.15 ± 5.47 ^b^
A+1370C	AA	20	49.19 ± 10.03 ^ab^	1.88 ± 0.84 ^ab^	22.02 ± 4.89
	AC	82	44.62 ± 12.34 ^a^	1.53 ± 1.08 ^a^	20.26 ± 5.39
	CC	95	50.50 ± 12.73 ^b^	2.19 ± 1.30 ^b^	22.34 ± 5.56
G+1373T	GG	18	48.74 ± 10.47 ^ab^	1.77 ± 0.81 ^ab^	21.51 ± 4.86
	GT	77	44.57 ± 12.41 ^a^	1.54 ± 1.09 ^a^	20.27 ± 5.42
	TT	102	50.30 ± 12.59 ^b^	2.17 ± 1.28 ^b^	22.31 ± 5.54

Note: Mean values in the same column with different superscripts (a, b) are significantly different (*p* < 0.05).

## Data Availability

Publicly available datasets were analyzed in this study. This data can be found here: [https://ftp.cngb.org/pub/CNSA/data2/CNP0001186/CNS0254395/CNA0014632//, accessed on 27 July 2023, accession number: MG787173].

## References

[1] Jin S., Fu Y., Hu Y., Fu H., Jiang S., Xiong Y., Qiao H., Zhang W., Gong Y., Wu Y. (2021). Identification of candidate genes from androgenic gland in *Macrobrachium nipponense* regulated by eyestalk ablation. Sci. Rep..

[2] (2013). Ministry of Agriculture, People’s Republic of China. http://www.moa.gov.cn/xw/bmdt/201904/t20190418_6194535.htm.

[3] Sun S., Xuan F., Ge X., Fu H., Zhu J., Zhang S. (2014). Identification of differentially expressed genes in hepatopancreas of oriental river prawn, *Macrobrachium nipponense* exposed to environmental hypoxia. Gene.

[4] Mangum C.P. (1997). Adaptation of the oxygen transport system to hypoxia in the blue crab, *Callinectes sapidus*. Am. Zool..

[5] McMahon B.R. (2001). Respiratory and circulatory compensation to hypoxia in crustaceans. Respir Physiol..

[6] Ma K., Feng J., Lin J., Li J. (2011). The complete mitochondrial genome of *Macrobrachium nipponense*. Gene.

[7] Cheng W., Liu C.H., Kuo C.M.J.A. (2003). Effects of dissolved oxygen on hemolymph parameters of freshwater giant prawn, *Macrobrachium rosenbergii* (de Man). Aquaculture.

[8] Yang M., Sun S., Fu H., Qiao H., Zhang W., Gong Y., Jiang S., Xiong Y., Xu L., Zhao C. (2019). Hypoxia and reoxygenation on antioxidant enzyme activities and histological structure of *Macrobrachium nipponense*. J. Fish. Sci. China.

[9] Xu L., Yang M., Fu H., Sun S., Wu Y. (2018). Molecular Cloning and Expression of *MnGST-1* and *MnGST-2* from Oriental River Prawn, *Macrobrachium nipponense*, in Response to Hypoxia and Reoxygenation. Int. J. Mol. Sci..

[10] Sun S., Xuan F., Fu H., Zhu J., Ge X., Gu Z. (2015). Transciptomic and histological analysis of hepatopancreas, muscle and gill tissues of oriental river prawn (*Macrobrachium nipponense*) in response to chronic hypoxia. BMC Genom..

[11] Frova C. (2006). Glutathione transferases in the genomics era: New insights and perspectives. Biomol. Eng..

[12] Zhou J., Wang W.-N., Wang A.-L., He W.-Y., Zhou Q.-T., Liu Y., Xu J. (2009). Glutathione S-transferase in the white shrimp Litopenaeus vannamei: Characterization and regulation under pH stress. Comp. Biochem. Physiol. Part C Toxicol. Pharmacol..

[13] Hayes J.D., Flanagan J.U., Jowsey I.R. (2005). Glutathione transferases. Annu. Rev. Pharmacol. Toxicol..

[14] Meister A. (1983). Transport and metabolism of glutathione and γ-glutamyl amino acids. Biochem. Soc. Trans..

[15] Meister A. (1988). Glutathione metabolism and its selective modification. J. Biol. Chem..

[16] Vignal A., Milan D., SanCristobal M., Eggen A. (2002). A review on SNP and other types of molecular markers and their use in animal genetics. Genet. Sel. Evol..

[17] Zhao Y., Wang H., Ji X., Zeng Y., Yang P., Ding L. (2010). Isolation and characterization of 20 polymorphic microsatellite markers in *Macrobrachium nipponense*. Conserv. Genet. Resour..

[18] Song K.-H., Kim W.J. (2011). Isolation and characterization of polymorphic microsatellites from the oriental river prawn *Macrobrachium nipponense* (Caridea: Palaemonidae). J. Crustac. Biol..

[19] Qiao H., Lv D., Jiang S., Sun S., Gong Y., Xiong Y., Jin S., Fu H. (2013). Genetic diversity analysis of oriental river prawn, *Macrobrachium nipponense*, in Yellow River using microsatellite marker. Evolution.

[20] Jombart T., Ahmed I. (2011). adegenet 1.3-1: New tools for the analysis of genome-wide SNP data. Bioinformatics.

[21] Muhammad S. (2014). Association mapping for salinity tolerance in cotton (*Gossypium hirsutum* L.) germplasm from US and diverse regions of China. Aust. J. Crop Sci..

[22] Lalitha S. (2000). Primer premier 5. Biotech Softw. Internet Rep..

[23] Jiang S., Xiong Y., Xia Z., Wang J., Zhang W., Cheng D., Gong Y., Wu Y., Qiao H., Fu H. (2022). Identification SNPs in vitellogenin gene and their association with ovarian development and growth of *Macrobrachium nipponense*. Aquac. Res..

[24] Tamura K., Stecher G., Kumar S. (2021). MEGA11: Molecular evolutionary genetics analysis version 11. Mol. Biol. Evol..

[25] Kuo C.-H., Janzen F.J. (2004). Genetic effects of a persistent bottleneck on a natural population of ornate box turtles (*Terrapene ornata*). Conserv. Genet..

[26] Nagy S., Poczai P., Cernák I., Gorji A.M., Hegedűs G., Taller J. (2012). PICcalc: An online program to calculate polymorphic information content for molecular genetic studies. Biochem. Genet..

[27] Allen P., Bennett K., Heritage B. (2014). SPSS Statistics Version 22: A Practical Guide.

[28] Slate J., Pemberton J.M. (2010). Admixture and patterns of linkage disequilibrium in a free-living vertebrate population. J. Evol. Biol..

[29] García-Guerrero M., Orduña-Rojas J., Cortés-Jacinto E. (2011). Oxygen Consumption of the Prawn Macrobrachium americanum over the Temperature Range of its Native Environment and in Relation to its Weight. N. Am. J. Aquac..

[30] Yin S.-W., Huang H., Lei C.-G., Chen G.-H., Zhang B. (2007). Application and Prospect of DNA Molecular Markers in Genetics and Breeding of Marine Fish. Hai Nan Da Xue Xue Bao.

[31] Xu Z., Taylor J.A. (2009). SNPinfo: Integrating GWAS and candidate gene information into functional SNP selection for genetic association studies. Nucleic Acids Res..

[32] Wang Z., Moult J. (2001). SNPs, protein structure, and disease. Hum. Mutat..

[33] Zhong S., Su Y., Mao Y., Liu M., Wang J., Zhang Q. (2018). Development and characterization of 24 SNP markers in Kuruma shrimp (*Marsupenaeus japonicus*) by illumina sequencing. Conserv. Genet. Resour..

[34] Jung H., Lyons R.E., Li Y., Thanh N.M., Dinh H., Hurwood D.A., Salin K.R., Mather P.B. (2014). A candidate gene association study for growth performance in an improved giant freshwater prawn (*Macrobrachium rosenbergii*) culture line. Mar. Biotechnol..

[35] Zeng D., Chen X., Li Y., Peng M., Ma N., Jiang W., Yang C., Li M.J. (2008). Analysis of Hsp70 in *Litopenaeus vannamei* and detection of SNPs. J. Crustac. Biol..

[36] Sun S., Fu H., Zhu J., Ge X., Wu X., Qiao H., Jin S., Zhang W. (2018). Molecular cloning and expression analysis of lactate dehydrogenase from the oriental river prawn *Macrobrachium nipponense* in response to hypoxia. Int. J. Mol. Sci..

[37] Xu L., Yang M., Fu H., Sun S., Qiao H., Zhang W., Gong Y., Jiang S., Xiong Y., Jin S. (2020). Molecular cloning, expression, and in situ hybridization analysis of MnGPx-3 and MnGPx-4 from oriental river prawn, *Macrobrachium nipponense*, in response to hypoxia and reoxygenation. PLoS ONE.

[38] Sun S., Gu Z., Fu H., Zhu J., Ge X., Xuan F. (2016). Molecular cloning, characterization, and expression analysis of p53 from the oriental river prawn, *Macrobrachium nipponense*, in response to hypoxia. Fish Shellfish. Immunol..

[39] Sun S., Xuan F., Fu H., Zhu J., Ge X., Wu X. (2017). Molecular cloning, characterization and expression analysis of caspase-3 from the oriental river prawn, *Macrobrachium nipponense* when exposed to acute hypoxia and reoxygenation. Fish Shellfish Immunol..

[40] Zhang W.-Y., Niu C.-J., Chen B.-J., Storey K.B. (2018). Digital Gene Expression Profiling reveals transcriptional responses to acute cold stress in Chinese soft-shelled turtle Pelodiscus sinensis juveniles. Cryobiology.

[41] Feng Y., Zhang D., Lv J., Gao B., Li J., Liu P. (2019). Identification of SNP markers correlated with the tolerance of low-salinity challenge in swimming crab (Portunus trituberculatus). Acta Oceanol. Sin..

[42] Morin P.A., Aitken N.C., Rubio-Cisneros N., Dizon A.E., Mesnick S. (2007). Characterization of 18 SNP markers for sperm whale (Physeter macrocephalus). Mol. Ecol. Notes.

[43] Wang W., Yi Q., Ma L., Zhou X., Zhao H., Wang X., Qi J., Yu H., Wang Z., Zhang Q. (2014). Sequencing and characterization of the transcriptome of half-smooth tongue sole (*Cynoglossus semilaevis*). BMC Genom..

[44] Cao T., Bai J., Yu L., Fan J.J. (2012). Polymorphisms of SNPs in ALDO B gene and association analysis with growth traits in grass carp (*Ctenopharyngodon idellus*). J. Fish. China.

[45] Montalban G., Bonache S., Moles-Fernández A., Gisbert-Beamud A., Tenés A., Bach V., Carrasco E., López-Fernández A., Stjepanovic N., Balmaña J. (2019). Screening of *BRCA1/2* deep intronic regions by targeted gene sequencing identifies the first germline *BRCA1* variant causing pseudoexon activation in a patient with breast/ovarian cancer. J. Med. Genet..

[46] Rhine C.L., Cygan K.J., Soemedi R., Maguire S., Murray M.F., Monaghan S.F., Fairbrother W.G. (2018). Hereditary cancer genes are highly susceptible to splicing mutations. PLoS Genet..

[47] Li J., Li Y., Ni H., Yang Z., Chen J., Li Y., Ding S., Jiang X., Wang M., Li L. (2020). A novel splice-site mutation in *MSH2* is associated with the development of lynch syndrome. Front. Oncol..

[48] Wang Y., Bernhardy A.J., Nacson J., Krais J.J., Tan Y.-F., Nicolas E., Radke M.R., Handorf E., Llop-Guevara A., Balmaña J. (2019). BRCA1 intronic Alu elements drive gene rearrangements and PARP inhibitor resistance. Nat. Commun..

[49] Reumers J., Conde L., Medina I., Maurer-Stroh S., Van Durme J., Dopazo J., Rousseau F., Schymkowitz J. (2008). Joint annotation of coding and non-coding single nucleotide polymorphisms and mutations in the SNPeffect and PupaSuite databases. Nucleic Acids Res..

[50] Slatkin M. (2008). Linkage disequilibrium—Understanding the evolutionary past and mapping the medical future. Nat. Rev. Genet..

[51] Jorde L.B. (1995). Linkage disequilibrium as a gene-mapping tool. Am. J. Hum. Genet..

